# Positive association of ethylene oxide levels with young stroke: a population-based study

**DOI:** 10.3389/fnagi.2024.1391176

**Published:** 2024-07-02

**Authors:** Lingying Le, Ziwei Lan, Chenxi Chen

**Affiliations:** ^1^Department of Neurology, West China Xiamen Hospital of Sichuan University, Xiamen, China; ^2^Department of Neurology, The Second Xiangya Hospital of Central South University, Changsha, China; ^3^Department of Plastic Surgery, Zhongshan Hospital (Xiamen), Fudan University, Xiamen, China

**Keywords:** ethylene oxide, stroke, epidemiology, NHANES, cross-sectional study

## Abstract

**Background:**

Ethylene oxide (EtO), a highly reactive organic compound with extensive industrial applications, poses significant health risks. The association between EtO exposure and stroke was not well established. This study examined the association between EtO exposure and stroke among US adults using data from the 2013–2018 National Health and Nutrition Examination Survey (NHANES).

**Methods:**

We used appropriately weighted multifactorial logistic regression models to analyze the data and validated the findings with smoothed curve fitting. Stratified analysis and interaction assessments were performed to evaluate the robustness of the findings.

**Results:**

The study included 5,071 participants, balanced between men and women, with a stroke prevalence of 4.1%. Higher EtO levels were associated with rising rates of stroke (OR = 1.23, 95% CI: 1.06–1.42). Individuals in the top 25% group displayed a stroke prevalence 1.6 times higher than those in the bottom 25% group (OR = 1.60, 95%CI: 1.03–2.48). Stratified analysis demonstrated a significant positive association between EtO and stroke in individuals under 50 years (OR = 1.94, 95%CI: 1.38–2.72), while no significant association was found in those aged 50 and above (OR = 0.97, 95%CI: 0.83–1.14).

**Conclusion:**

This study identified a significant association between EtO exposure and stroke occurrence in young adults in the United States.

## Introduction

Stroke ranked among the leading causes of death globally, affecting millions each year through progressive blood vessel blockages that disrupted neurological function (Kuriakose and Xiao, 2020). It was the second most common cause of death worldwide, following ischemic heart disease, and the third leading cause of death and disability (2021; Owolabi et al., 2022). As the population aged, the prevalence of stroke continued to rise (Roth et al., 2020). Stroke imposed a significant global public health burden, with economic losses exceeding $721 billion, or 0.66% of the global Gross Domestic Product (GDP) (2022). Therefore, preventing and managing stroke risk factors were crucial.

Increasing evidence indicated that environmental pollution may be associated with the onset and progression of stroke (Verhoeven et al., 2021; Tian et al., 2022; Poulsen et al., 2023; Ranta et al., 2023). Ethylene oxide (EtO) was a highly active organic compound with extensive industrial applications, posing significant hazards to human health. EtO was widely used in the manufacture of various synthetic products (Lynch et al., 2022) such as ethylene glycol, synthetic detergents, emulsifiers, nonionic surfactants, antifreeze, plasticizers, lubricants, pesticides, and more (Kirman et al., 2021). Additionally, EtO was a broad-spectrum, high-efficiency bactericidal disinfectant, commonly employed in medical disinfection and industrial sterilization (Mendes et al., 2007). It was found in textiles, leather, precision instruments, biological products, medical instruments, rubber products, and various gas fumigation disinfection processes. Moreover, it was prevalent in our living environment, present in polluted air, automobile exhaust, and tobacco smoke (Bono et al., 2002). EtO could be absorbed through the respiratory tract and skin, distributed to various tissues and organs throughout the body, and caused harm (Filser and Klein, 2018).

Previous studies on EtO have primarily focused on its carcinogenicity. Numerous investigations unequivocally established EtO’s carcinogenic properties (O'Kelley et al., 2023), particularly its link to lympho-hematopoietic cancer and breast cancer (Jinot et al., 2018; Marsh et al., 2019). As a result, the International Agency for Research on Cancer (IARC) classified EtO as a Group 1 human carcinogen (Vincent et al., 2019). In recent years, the COVID-19 pandemic significantly increased the general population’s exposure to EtO through sterilized medical protective equipment. This prompted scholars to explore the potential effects of chronic EtO exposure on public health (Jain, 2020; Huang et al., 2023; Wu et al., 2024).

Emerging evidence indicated that chronic EtO exposure was significantly associated with various risk factors for vascular diseases, such as hypertension (Ningtao et al., 2022), diabetes (Jingyu et al., 2021), hyperlipidemia (Xu et al., 2022), and smokers (Brandon et al., 2021). Additionally, recent studies showed that EtO exposure may impair cardiovascular health by inducing oxidative stress and inflammatory responses (Oluwatobi et al., 2021), damaging endothelial cells (Mahmood et al., 2021), and increasing vascular stiffness (Viviane and Peter, 2021). These findings suggested that EtO may also be involved in cerebrovascular diseases such as stroke. Considering the growing concern about environmental pollution’s impact on public health and the lack of studies on EtO’s association with stroke, this study aimed to explore the potential correlation between EtO exposure and stroke risk using data from the National Health and Nutrition Examination Survey (NHANES). This research strived to offer new insights into the environmental risk factors for stroke and contribute to the development of policies to mitigate these risks.

## Materials and methods

### Study population

**Figure 1:**
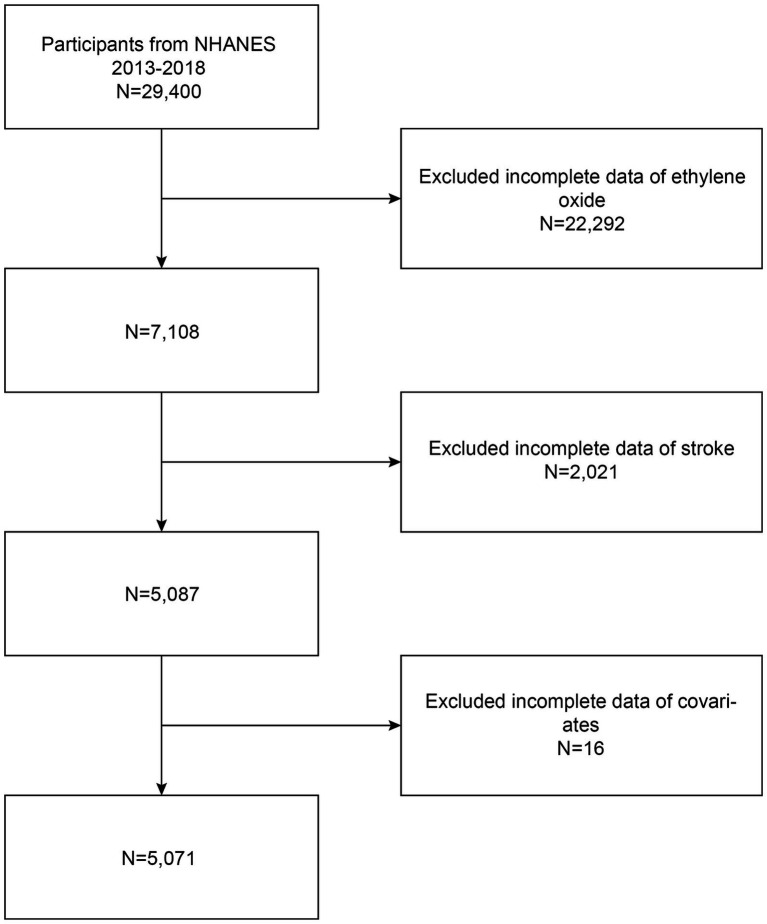
Flowchart of the sample selection from NHANES 2013–2018.

The study analyzed data from the National Health and Nutrition Examination Survey (NHANES), conducted by the Centers for Disease Control and Prevention (CDC) among non-institutionalized U.S. residents (Borrud et al., 2014). Initiated in 1999, this survey collects nationally representative data using a scientifically rigorous methodology, including demographic information, health examination data, and interview reports. Data from the 2013–2018 cycles, encompassing 29,400 participants, were included. After excluding 24,313 subjects with missing or unknown data on hemoglobin adducts of ethylene oxide (HbEtO) and stroke, and 16 participants with missing covariates, the final dataset comprised 5,071 adults. Figure 1 depicts the detailed screening process. The National Center for Health Statistics (NCHS) Ethics Review Committee approved this study, and all participants provided written informed consent.

### Definition of research variables

#### Measurement of ethylene oxide

EtO is challenging to detect due to its short biological half-life of approximately 42 min (Filser, 1992). However, EtO reacts with valine in hemoglobin to form HbEtO, which has a half-life of about 4 months and serves as an effective biomarker for EtO exposure (Song et al., 2023). Therefore, this study measured EtO levels by detecting HbEtO. Blood samples were collected from fasting participants and analyzed at the National Center for Environmental Health (NCEH) using high performance liquid chromatography–tandem mass spectrometry (HPLC-MS/MS). The results were expressed as pmol adduct per gram of hemoglobin, and the procedure adhered to NCEH quality control standards. The EtO level was used as our exposure variable.

#### Assessment of stroke

The diagnosis of stroke was determined through self-reported responses in a personal interview using the NHANES medical conditions questionnaire. Participants were queried about whether a physician or other healthcare practitioner had ever diagnosed them with a stroke. Individuals who responded affirmatively were categorized as having experienced a stroke. This result was used as the outcome variable.

#### Covariates

Covariates included gender, age, race/ethnicity, education level, marital status, body mass index (BMI, kg/m^2^), low-density lipoprotein cholesterol (LDL-C, mmol/L). Categorize race as Mexican American, Non-Hispanic Black, Non-Hispanic White, Other Hispanic, and Other Races. Categorized educational attainment as below high school, high school diploma, or above high school diploma. Categorized marital status as married or living with a partner, or living alone. Pre-existing conditions (hypertension, diabetes, and cancer) were determined based on a questionnaire asking whether the condition was notified. The calculation of body mass index (BMI) involved dividing an individual’s weight by the square of their height. For comprehensive testing principles and procedures, please refer to the NHANES website at https://www.cdc.gov/nchs/nhanes/.

### Statistical analysis

All statistical procedures were completed using EmpowerStats^1^ and R 3.4.3 software.^2^ Since HbEtO data were skewed, they were ln-transformed and grouped into quartiles for analysis. The mean ± standard deviation (SD) was used to represent continuous variables, while ratios were employed to represent categorical variables. The Kruskal-Wallis rank sum test assessed differences in continuous variables, while the chi-square test assessed differences in categorical variables. Multiple logistic regression analyses were used to examine the relationship between EtO exposure and stroke, employing three models: Model 1 (crude model), Model 2 (adjusted for main demographic variables), and Model 3 (further adjusted for education, marital status, BMI, LDL-C, hypertension, diabetes, and cancer history). The association was validated using smoothed curve fitting. Subgroup analysis and interaction testing provided insights into the association across demographic categories. *p* values below 0.05 were considered statistically significant.

## Results

### Baseline characteristics of participants

Figure 1 presents a flowchart illustrating the process of screening participants. The study included 5,071 participants, averaging 50.03 ± 17.46 years in age. Of these, 2,494 were older than 50, with a near-even gender split of 49.22% male and 50.78% female. Participants were grouped into quartiles based on their ln-transformed HbEtO levels (quartile 1: 1.76–2.76; quartile 2: 2.77–3.09; quartile 3: 3.10–3.77; quartile 4: 3.78–7.29). The overall prevalence of stroke was 4.1%, with the highest prevalence in quartile 3 at 4.65%, but the differences between the groups were not statistically significant (*p* = 0.439). There were significant differences between quartiles of ln-HbEtO in terms of age, gender, race, education level, marital status, diabetes, cancer, and BMI (all *p* < 0.05). Table 1 provides detailed information on the baseline characteristics of the participants.

**Table 1:** Basic characteristics of participants by hemoglobin adducts of ethylene oxide levels among U.S. adults.

Characteristics	Ln-transformed HbEtO				*p*-value
	Q1 (*N* = 1,263)	Q2 (*N* = 1,271)	Q3 (*N* = 1,269)	Q4 (*N* = 1,268)	
Age (years)	50.60 ± 17.77	51.58 ± 18.02	51.38 ± 17.49	46.56 ± 16.01	<0.001
Gender, (%)					<0.001
Male	44.73	46.89	46.57	58.68	
Female	55.27	53.11	53.43	41.32	
Race/ethnicity, (%)					<0.001
Mexican American	14.96	19.51	16.71	8.68	
Other Hispanic	12.27	12.82	9.85	6.62	
Non-Hispanic White	45.61	36.51	27.90	39.98	
Non-Hispanic Black	15.28	16.76	20.49	30.52	
Other Races	11.88	14.40	25.06	14.20	
Education level, (%)					<0.001
Less than high school	17.10	21.16	20.88	26.97	
High school or GED	20.90	20.22	22.14	29.97	
Above high school	62.00	58.62	56.97	43.06	
Marital status, (%)					<0.001
Married/living with partner	63.34	63.81	62.73	50.47	
Live alone	36.66	36.19	37.27	49.53	
Hypertension, (%)					0.207
Yes	37.37	34.78	38.38	38.25	
No	62.63	65.22	61.62	61.75	
Diabetes, (%)					<0.001
Yes	11.80	14.16	18.60	12.15	
No	88.20	85.84	81.40	87.85	
Cancer, (%)					0.036
Yes	11.80	9.76	9.06	8.60	
No	88.20	90.24	90.94	91.40	
BMI (kg/m^2^)	30.48 ± 7.51	30.16 ± 7.36	29.03 ± 6.77	28.28 ± 6.67	<0.001
LDL-C (mmol/L)	2.82 ± 0.59	2.85 ± 0.65	2.84 ± 0.63	2.82 ± 0.65	0.657
Stroke, (%)					0.439
Yes	3.48	3.86	4.65	4.42	
No	96.52	96.14	95.35	95.58	

Mean ± SD for continuous variables: the *p* value was calculated by the weighted linear regression model; (%) for categorical variables: the *p* value was calculated by the weighted chi-square test.HbEtO, hemoglobin adducts of ethylene oxide; Q, quartile; GED, general educational development; BMI, body mass index; LDL-C, low-density lipoprotein.

### Association between hemoglobin adducts of ethylene oxide and stroke

**Figure 2:**
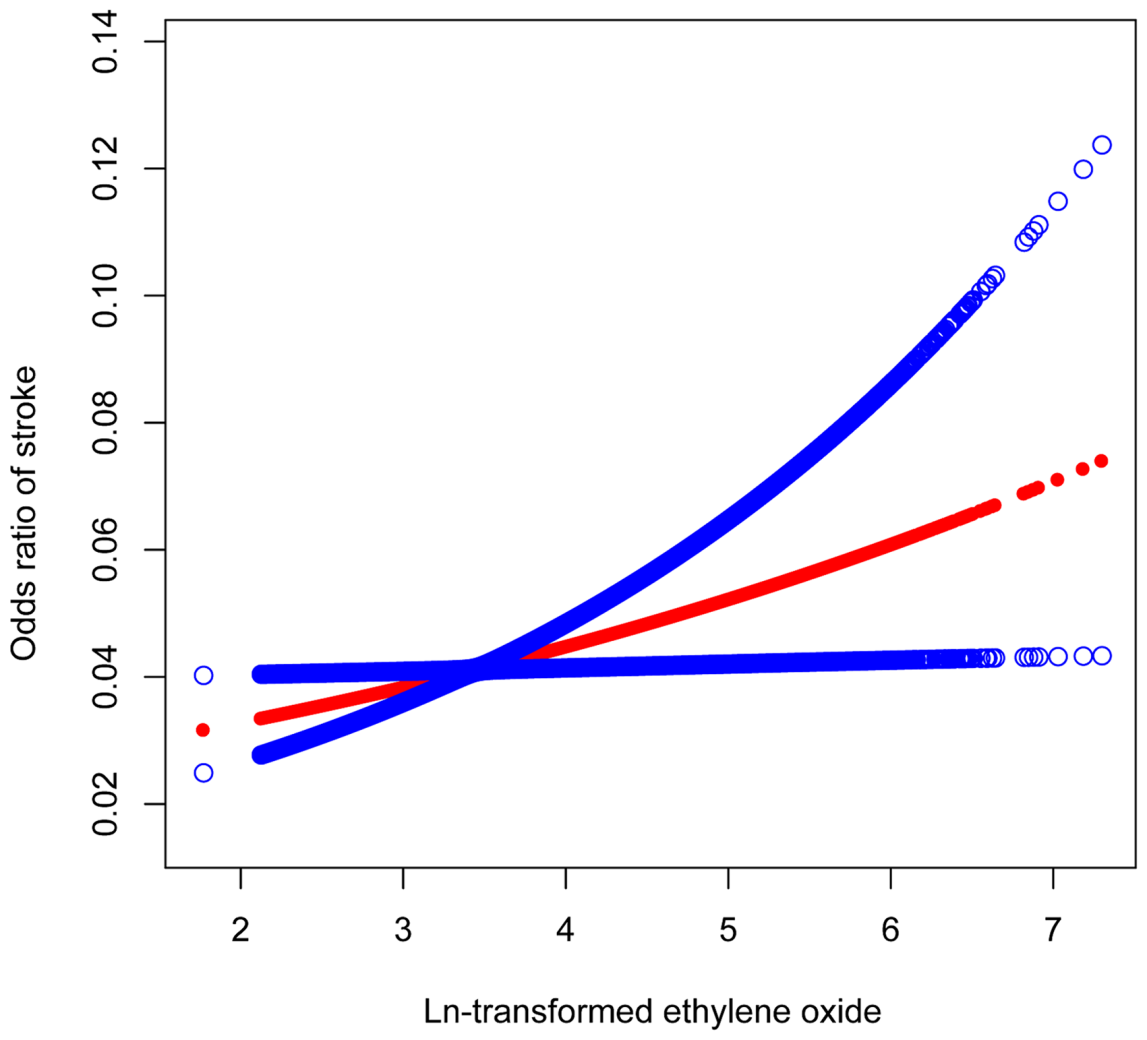
The relationship between ln-transformed ethylene oxide and stroke. The solid red line represents the smooth curve fit between variables. Blue bands represent the 95% of confidence interval from the fit.

The results of the multifactor regression analysis for the three models are presented in Table 2. In the unadjusted crude model 1, the association between HbEtO and stroke was not significant (OR: 1.12, 95% CI: 0.99–1.27). However, model 2, which adjusted for demographic variables including age, gender, and race, showed a significant positive correlation between HbEtO and stroke (OR: 1.27, 95% CI: 1.11–1.45). This positive association remained significant in model 3, which further adjusted for all covariates. Each 1-unit increase in ln-HbEtO was associated with a 23% increased risk of stroke (OR: 1.23, 95% CI: 1.06–1.42). When ln-HbEtO was divided into quartiles, the risk of stroke in the highest quartile in the fully adjusted model was 1.6 times that of participants in the lowest quartile (OR: 1.60, 95% CI: 1.03–2.48). To elucidate the correlation between HbEtO and stroke, we plotted a smoothed curve fit (Figure 2), which graphically depicted the positive association.

**Table 2:** Multiple logistic regression associations of ln-transformed HbEtO with stroke.

	OR (95% CI)		
	Crude model (Model 1)	Minimally adjusted model (Model 2)	Fully adjusted model (Model 3)
Continuous ln-transformed EO	1.12 (0.99, 1.27)	1.27 (1.11, 1.45)	1.23 (1.06, 1.42)
Categories			
Quartile 1	1 (Reference)	1 (Reference)	1 (Reference)
Quartile 2	1.11 (0.73, 1.68)	1.06 (0.70, 1.63)	1.07 (0.70, 1.64)
Quartile 3	1.35 (0.91, 2.01)	1.39 (0.92, 2.09)	1.33 (0.88, 2.02)
Quartile 4	1.28 (0.86, 1.91)	1.75 (1.14, 2.67)	1.60 (1.03, 2.48)
*P* for trend	0.30	0.01	0.03

Model 1: no covariates were adjusted. Model 2: age, gender, and race were adjusted. Model 3: age, gender, race, education level, marital status, BMI, hypertension, diabetes, cancer and LDL-C were adjusted.HbEtO, hemoglobin adducts of ethylene oxide; OR, odds ratio; 95% CI, 95% confidence interval; BMI, body mass index; LDL-C, low-density lipoprotein-cholesterol.

### Subgroup analysis

To explore the consistency of the relationship between HbEtO and stroke across different populations and identify potential population-specific parameters, we performed subgroup analysis and interaction assessments (Table 3). We found inconsistent associations when stratified by age. The positive correlation between HbEtO and stroke was significant in the 20–50 age group (OR: 1.94, 95% CI: 1.38–2.72) but not in the over 50 age group (OR: 0.97, 95% CI: 0.83–1.14). Additionally, no significant interactions were detected in other stratified analyses, indicating that the association between HbEtO and stroke was independent of gender, education level, marital status, BMI, hypertension, diabetes, and cancer (all P for interaction >0.05).

**Table 3:** Subgroup analysis of the association between ln-transformed HbEtO and stroke.

Subgroup	Stroke [OR (95%CI)]	*P* for interaction
Gender		0.548
Male	1.18 (0.98, 1.42)	
Female	1.09 (0.88, 1.34)	
Age		<0.001
20–50 years	1.94 (1.38, 2.72)	
> 50 years	0.97 (0.83,1.14)	
Education level		0.878
Less than high school	1.12 (0.88, 1.44)	
High school or GED	1.07 (0.82, 1.38)	
Above high school	1.17 (0.93,1.46)	
Marital status		0.373
Married/living with partner	1.21 (1.00, 1.46)	
Live alone	1.07 (0.88, 1.30)	
BMI		0.651
< 24.9 kg/m^2^	1.15 (0.84, 1.56)	
25–29.9 kg/m^2^	1.24 (0.99, 1.55)	
≥ 30 kg/m^2^	1.07 (0.85, 1.34)	
Hypertension		0.999
Yes	1.13 (0.96, 1.33)	
No	1.13 (0.88, 1.45)	
Diabetes		0.541
Yes	1.07 (0.80, 1.41)	
No	1.18 (1.00, 1.39)	
Cancer		0.269
Yes	0.94 (0.65, 1.35)	
No	1.16 (1.00, 1.36)	

Age, gender, race, education level, marital status, BMI, hypertension, diabetes, cancer and LDL-C were adjusted.HbEtO, hemoglobin adducts of ethylene oxide; OR, odds ratio; 95% CI, 95% confidence interval; GED, general educational development; BMI, body mass index; LDL-C, low-density lipoprotein-cholesterol.

## Discussion

In our cross-sectional study involving 5,071 individuals representative of the general U.S. population, we found a positive association between EtO and stroke. This association persisted across all subgroup analyses except for age, suggesting the results’ reliability. This finding provided new evidence linking environmental pollutants to stroke occurrence.

Notably, this study was the first to report a positive correlation between EtO exposure and stroke. Ethylene oxide is known to pose significant risks to human health. Historically, research has primarily focused on its carcinogenicity (Thier and Bolt, 2000; Jones et al., 2023), often highlighting the hazards associated with occupational exposure (Sheikh, 1984; Park, 2020). Recent studies have also established a strong association between EtO and cardiovascular diseases in the general U.S. population. Research showed that EtO negatively impacted the cardiovascular system, influencing blood pressure (Ningtao et al., 2022) and contributing to systemic inflammation and altered blood lipid levels (Xu et al., 2022). These findings align with previous studies suggesting that long-term EtO exposure increased cardiovascular disease risk (Guowei et al., 2021). A recent large-sample cross-sectional study, which combined various indicators including sleep duration, diet, body mass index, blood glucose, blood pressure, lipids, and smoking status, using Cox regression and linear regression models, found a significant association between EtO exposure and adverse cardiovascular outcomes (Ruijie et al., 2023). Collectively, these studies indicated that ethylene oxide, in addition to being a known carcinogen, may increase the risk of cardiovascular diseases through multiple mechanisms.

The impact of environmental pollution on stroke has gained significant attention recently, with growing evidence linking pollution to cerebrovascular diseases (Tian et al., 2022). Suggestions from a meta-analysis (de Bont et al., 2022) that included 56 publications suggested that short-term exposure to PM2.5, PM10, and NOx was correlated with an increased prevalence of stroke, and that prolonged exposure was correlated with an elevated likelihood of stroke mortality, particularly among older individuals, individuals with heart conditions, and individuals with higher body weight. As the population ages, preventing and controlling environmental factors related to stroke becomes critical. Given the significant association between cardiovascular disease and EtO exposure, our study utilized nationally representative data from the NHANES database to investigate the potential correlation between cerebrovascular disease (stroke) and EtO exposure, aiming to understand its impact on the central nervous system better.

In this investigation, higher ln-HbEtO levels were associated with a higher stroke rate, as determined by a fully adjusted logistic regression model. Our subgroup analysis revealed that the positive relationship between EtO levels and stroke remained significant for U.S. adults younger than 50 years, while no association was found in those over 50. This suggests a noteworthy correlation between EtO levels and stroke among younger individuals.

The biological mechanisms linking EtO exposure to stroke were unclear, and it may be involved in the mechanisms of stroke through multiple pathways. Studies on animals have demonstrated that prolonged exposure to EtO can trigger oxidative stress (Somade et al., 2021), enhance lipid peroxidation, and disrupt the glutathione redox cycle (Katoh et al., 1988). This results in the overproduction of reactive oxygen species, activation of inflammatory pathways (Xu et al., 2022), and upregulation of inflammatory cytokines (Guowei et al., 2021), leading to vascular endothelial cell damage and the development of atherosclerotic plaques (Rocha and Libby, 2009). Additionally, EtO exposure caused endothelial dysfunction (Adedara and Farombi, 2010), which manifests as impaired endothelium-dependent vasodilation and reduced endothelial cell proliferation. This disruption of vascular tone and increased vascular stiffness interfered with the normal systolic-diastolic response of cerebral blood vessels (Mahmood et al., 2021), impairing cerebral vascular autoregulation and elevating stroke risk. Furthermore, EtO exposure has been associated with increased platelet aggregation and a tendency for thrombosis by influencing platelet activation and coagulation factor expression, which may also contribute to an increased risk of stroke (Yixuan et al., 2024).

There was no uniform definition of young stroke. Based on previous studies, young stroke was generally defined as stroke occurring in adults younger than 50 years (Ekker et al., 2018). Studies have shown a higher proportion of unexplained strokes in young adults compared to older adults (Maaijwee et al., 2014). This suggests that the etiology of young stroke was more complex. The results of our subgroup analysis suggested that a more significant positive association between ethylene oxide and stroke in young may have several seemingly plausible explanations. First, the age of 20–50 years was a period of more frequent occupational and social activities, and there may be more exposure to environmental pollutants such as automobile exhaust and industrial emissions, which increased the chance of dermal contact and respiratory inhalation of EtO, leading to increased exposure to EtO in the body, making the correlation with stroke more significant. Second, the metabolic functions of the liver and kidneys were robust in young adulthood, and the increased amount of reactive oxygen species and free radicals generated during the metabolism and clearance of ethylene oxide may lead to organ degeneration and increased oxidative damage (Filser and Klein, 2018). It has been shown that increased chronic oxidative stress was a deleterious factor contributing to the development of insulin resistance, dyslipidemia, and other stroke risk factors (Tangvarasittichai, 2015), which indirectly contributed to the increased prevalence of stroke. In addition, aging was a physiological process that continues over time (Dziechciaż and Filip, 2014), and some studies have shown that significant vascular aging features including aortic elastin fiber breaks and collagen deposition were observed in older individuals (Zhang et al., 2023), and that there were differences in the sensitivity of vascular endothelial cells at different ages, and that young adults were more sensitive to oxidative stress, inflammatory responses, and cytotoxic effects induced by ethylene oxide (Lynch et al., 1984). Therefore, the relationship between ethylene oxide and stroke was more prominent. Simultaneously, there were more risk factors for stroke in the elderly population, so that the role of EtO may be relatively masked. Further research is necessary to validate the findings of this study and to clarify the specific biological pathways involved.

This study has limitations. First, we were unable to determine a causal relationship between EtO exposure and stroke because it was a cross-sectional study, and longitudinal cohort studies are required for further validation. Second, there were limitations in the EtO biomarkers used, necessitating the development of more accurate measures. Third, despite including covariates and validating models with stratified analyses, we could not completely eliminate all confounding factors. Additionally, the reliance on self-reported data for stroke inclusion criteria in the NHANES database, without differentiation of stroke subtypes, limited our ability to accurately assess the subtypes of stroke. Despite these limitations, our investigation possesses notable advantages. We employed a large sample size of the general U.S. population sample, which was highly representative. Meanwhile, because of our extensive sample size, we had the opportunity to conduct subgroup analyses of various factors while controlling for many important confounders to ensure the reliability of our results.

## Conclusion

Our study conclusively shows a positive association between EtO levels and the incidence of young stroke in the general adult population in the United States. This finding provides a new perspective on environmental risk factors for young stroke, supporting the need for environmental policies to reduce pollutants and raising awareness about the health risks of environmental pollution among young adults.

Future research should explore the potential impact of reducing EtO exposure on stroke incidence and how other environmental and lifestyle factors may interact with EtO exposure to influence stroke risk. These directions will provide important insights for the development of effective stroke prevention strategies.

## Data availability statement

The raw data supporting the conclusions of this article will be made available by the authors, without undue reservation.

## Ethics statement

The studies involving humans were approved by the National Center for Health Statistics Research Ethics Review Board. The studies were conducted in accordance with the local legislation and institutional requirements. The participants provided their written informed consent to participate in this study.

## Author contributions

LL: Writing – original draft, Writing – review & editing. ZL: Writing – original draft. CC: Writing – review & editing.

## Footnotes

^1^ http://www.empowerstats.net/cn/index.php

^2^ https://www.r-project.org/

## References

[ref1] Adedara I. Farombi E. (2010). Induction of oxidative damage in the testes and spermatozoa and hematotoxicity in rats exposed to multiple doses of ethylene glycol monoethyl ether. Hum. Exp. Toxicol. 29, 801–812. doi: 10.1177/0960327109360115 20172899

[ref2] Bono R. Vincenti M. Saglia U. Pignata C. Russo R. Gilli G. (2002). Tobacco smoke and formation of N-(2-hydroxyethyl)valine in human hemoglobin. Arch. Environ. Health 57, 416–421. doi: 10.1080/00039890209601430 12641182

[ref3] Borrud L. Chiappa M. Burt V. Gahche J. Zipf G. Johnson C.. (2014). National Health and nutrition examination survey: national youth fitness survey plan, operations, and analysis, 2012. Vital Health Stat, 2, 1–24. 24709592

[ref9001] Brandon M. K. Caitlyn M. Luyu Z. Wanzhe Z. Deepak B. Víctor R. D. J.. (2021). Characterization of the association between cigarette smoking intensity and urinary concentrations of 2-hydroxyethyl mercapturic acid among exclusive cigarette smokers in the National Health and Nutrition Examination Survey (NHANES) 2011-2016. Biomarkers 26, 656–664. doi: 10.1080/1354750x.2021.1970809, PMID: 34409911 PMC8517914

[ref4] de Bont J. Jaganathan S. Dahlquist M. Persson Å. Stafoggia M. Ljungman P. (2022). Ambient air pollution and cardiovascular diseases: An umbrella review of systematic reviews and meta-analyses. J. Intern. Med. 291, 779–800. doi: 10.1111/joim.13467, PMID: 35138681 PMC9310863

[ref5] Dziechciaż M. Filip R. (2014). Biological psychological and social determinants of old age: bio-psycho-social aspects of human aging. Ann. Agric. Environ. Med. 21, 835–838. doi: 10.5604/12321966.1129943, PMID: 25528930

[ref6] Ekker M. Boot E. Singhal A. Tan K. Debette S. Tuladhar A.. (2018). Epidemiology, aetiology, and management of ischaemic stroke in young adults. Lancet Neurol. 17, 790–801. doi: 10.1016/s1474-4422(18)30233-3 30129475

[ref7] Feigin V. L. Brainin M. Norrving B. (2022). Corrigendum to: world stroke organization (WSO): global stroke fact sheet 2022. Int. J. Stroke 17:478. doi: 10.1177/17474930221080343 34986727

[ref8] Filser J. (1992). The closed chamber technique--uptake, endogenous production, excretion, steady-state kinetics and rates of metabolism of gases and vapors. Arch. Toxicol. 66, 1–10. doi: 10.1007/bf02307263 1580790

[ref9] Filser J. Klein D. (2018). A physiologically based toxicokinetic model for inhaled ethylene and ethylene oxide in mouse, rat, and human. Toxicol. Lett. 286, 54–79. doi: 10.1016/j.toxlet.2017.07.896, PMID: 28774830

[ref11] Guowei Z. Qi Z. Xiaowei W. Kai-Hong W. (2021). Association between blood ethylene oxide levels and the risk of cardiovascular diseases in the general population. Environ. Sci. Pollut. Res. Int. 28, 64921–64928. doi: 10.1007/s11356-021-15572-0, PMID: 34322816

[ref12] Huang Q. Li S. Wan J. Nan W. He B. (2023). Association between ethylene oxide exposure and prevalence of COPD: evidence from NHANES 2013-2016. Sci. Total Environ. 885:163871. doi: 10.1016/j.scitotenv.2023.163871 37149189

[ref13] Jain R. (2020). Associations between observed concentrations of ethylene oxide in whole blood and smoking, exposure to environmental tobacco smoke, and cancers including breast cancer: data for US children, adolescents, and adults. Environ. Sci. Pollut. Res. Int. 27, 20912–20919. doi: 10.1007/s11356-020-08564-z 32249385

[ref14] Jingyu G. Zhenzhen W. Guanglin C. An P. Gang L. (2021). Association of exposure to ethylene oxide with risk of diabetes mellitus: results from NHANES 2013-2016. Environ. Sci. Pollut. Res. Int. 28, 68551–68559. doi: 10.1007/s11356-021-15444-7, PMID: 34273079

[ref15] Jinot J. Fritz J. Vulimiri S. Keshava N. (2018). Carcinogenicity of ethylene oxide: key findings and scientific issues. Toxicol. Mech. Methods 28, 386–396. doi: 10.1080/15376516.2017.1414343, PMID: 29210319 PMC10883472

[ref16] Jones R. Fisher J. Medgyesi D. Buller I. Liao L. Gierach G.. (2023). Ethylene oxide emissions and incident breast cancer and non-Hodgkin lymphoma in a US cohort. J. Natl. Cancer Inst. 115, 405–412. doi: 10.1093/jnci/djad004, PMID: 36633307 PMC10086621

[ref17] Katoh T. Higashi K. Inoue N. Tanaka I. (1988). Effects of chronic inhalation of ethylene oxide on lipid peroxidation and glutathione redox cycle in rat liver. Res. Commun. Chem. Pathol. Pharmacol. 61, 281–284, PMID: 3187196

[ref19] Kirman C. Li A. Sheehan P. Bus J. Lewis R. Hays S. (2021). Ethylene oxide review: characterization of total exposure via endogenous and exogenous pathways and their implications to risk assessment and risk management. J. Toxicol. Environ. Health B Crit. Rev. 24, 1–29. doi: 10.1080/10937404.2020.1852988, PMID: 33323046

[ref20] Kuriakose D. Xiao Z. (2020). Pathophysiology and treatment of stroke: present status and future perspectives. Int. J. Mol. Sci. 21:7609. doi: 10.3390/ijms21207609, PMID: 33076218 PMC7589849

[ref21] Lynch H. Kozal J. Russell A. Thompson W. Divis H. Freid R.. (2022). Systematic review of the scientific evidence on ethylene oxide as a human carcinogen. Chem. Biol. Interact. 364:110031. doi: 10.1016/j.cbi.2022.110031, PMID: 35779612

[ref22] Lynch D. Lewis T. Moorman W. Burg J. Groth D. Khan A.. (1984). Carcinogenic and toxicologic effects of inhaled ethylene oxide and propylene oxide in F344 rats. Toxicol. Appl. Pharmacol. 76, 69–84. doi: 10.1016/0041-008x(84)90030-9, PMID: 6484993

[ref23] Maaijwee N. Rutten-Jacobs L. Schaapsmeerders P. van Dijk E. de Leeuw F. (2014). Ischaemic stroke in young adults: risk factors and long-term consequences. Nat. Rev. Neurol. 10, 315–325. doi: 10.1038/nrneurol.2014.72 24776923

[ref24] Mahmood R. Arif M. Muhammad A. B. A. Rabia M. Ujala A. Sulayman W.. (2021). Phytochemical analysis and protective effects of *Vaccinium macrocarpon* (cranberry) in rats (*Rattus norvegicus*) following ethylene oxide-induced oxidative insult. Bioengineered 12, 4593–4604. doi: 10.1080/21655979.2021.1955528, PMID: 34346287 PMC8806514

[ref25] Marsh G. Keeton K. Riordan A. Best E. Benson S. (2019). Ethylene oxide and risk of lympho-hematopoietic cancer and breast cancer: a systematic literature review and meta-analysis. Int. Arch. Occup. Environ. Health 92, 919–939. doi: 10.1007/s00420-019-01438-z, PMID: 31111206

[ref26] Mendes G. Brandão T. Silva C. (2007). Ethylene oxide sterilization of medical devices: a review. Am. J. Infect. Control 35, 574–581. doi: 10.1016/j.ajic.2006.10.014, PMID: 17980234

[ref27] Ningtao W. Wenya C. Yuxing W. Xiaoqing L. (2022). Association between blood ethylene oxide levels and the prevalence of hypertension. Environ. Sci. Pollut. Res. Int. 29, 76937–76943. doi: 10.1007/s11356-022-21130-z, PMID: 35668269

[ref9002] Oluwatobi T. S. Babajide O. A. Olubisi E. Anuoluwapo A. Ridwan O. A. (2021). Oxidative stress-mediated induction of pulmonary oncogenes, inflammatory, and apoptotic markers following time-course exposure to ethylene glycol monomethyl ether in rats. Metabol Open, 9, 100075., PMID: 33409483 10.1016/j.metop.2020.100075 PMC7773962

[ref28] O'Kelley L. Swanson B. Bishop-Royse J. (2023). Integrative literature review: ethylene oxide exposure signs and symptoms. Public Health Nurs. 40, 790–809. doi: 10.1111/phn.13216, PMID: 37254592

[ref29] Owolabi M. Thrift A. Mahal A. Ishida M. Martins S. Johnson W.. (2022). Primary stroke prevention worldwide: translating evidence into action. Lancet Public Health 7, e74–e85. doi: 10.1016/s2468-2667(21)00230-9, PMID: 34756176 PMC8727355

[ref30] Park R. (2020). Associations between exposure to ethylene oxide, job termination, and cause-specific mortality risk. Am. J. Ind. Med. 63, 577–588. doi: 10.1002/ajim.23115, PMID: 32378753 PMC7667669

[ref31] Poulsen A. Sørensen M. Hvidtfeldt U. Ketzel M. Christensen J. Brandt J.. (2023). Air pollution and stroke; effect modification by sociodemographic and environmental factors. A cohort study from Denmark. Int. J. Hyg. Environ. Health 251:114165. doi: 10.1016/j.ijheh.2023.114165 37121155

[ref32] Ranta A. Ozturk S. Wasay M. Giroud M. Béjot Y. Reis J. (2023). Environmental factors and stroke: risk and prevention. J. Neurol. Sci. 454:120860. doi: 10.1016/j.jns.2023.120860 37944211

[ref33] Rocha V. Libby P. (2009). Obesity, inflammation, and atherosclerosis. Nat. Rev. Cardiol. 6, 399–409. doi: 10.1038/nrcardio.2009.55, PMID: 19399028

[ref34] Roth G. Mensah G. Johnson C. Addolorato G. Ammirati E. Baddour L.. (2020). Global burden of cardiovascular diseases and risk factors, 1990-2019: update from the GBD 2019 study. J. Am. Coll. Cardiol. 76, 2982–3021. doi: 10.1016/j.jacc.2020.11.010, PMID: 33309175 PMC7755038

[ref35] Ruijie X. Linjian L. Changxiong L. Songlin X. Xiongjie H. Ya Z. (2023). Associations of ethylene oxide exposure and "Life's essential 8". Environ. Sci. Pollut. Res. Int. 30, 121150–121160. doi: 10.1007/s11356-023-30741-z, PMID: 37950781

[ref36] Sheikh K. (1984). Adverse health effects of ethylene oxide and occupational exposure limits. Am. J. Ind. Med. 6, 117–127. doi: 10.1002/ajim.4700060206 6380275

[ref37] Somade O. T. Ajayi B. O. Adeyi O. E. Adeshina A. A. Adekoya M. O. Abdulhameed R. O. (2021). Oxidative stress-mediated induction of pulmonary oncogenes, inflammatory, and apoptotic markers following time-course exposure to ethylene glycol monomethyl ether in rats. Metabol. Open 9:100075. doi: 10.1016/j.metop.2020.100075, PMID: 33409483 PMC7773962

[ref38] Song W. Hu H. Ni J. Zhang H. Zhang H. Yang G.. (2023). The relationship between ethylene oxide levels in hemoglobin and the prevalence of kidney stones in US adults: an exposure-response analysis from NHANES 2013-2016. Environ. Sci. Pollut. Res. Int. 30, 26357–26366. doi: 10.1007/s11356-022-24086-2, PMID: 36367648

[ref39] Tangvarasittichai S. (2015). Oxidative stress, insulin resistance, dyslipidemia and type 2 diabetes mellitus. World J. Diabetes 6, 456–480. doi: 10.4239/wjd.v6.i3.456, PMID: 25897356 PMC4398902

[ref40] Thier R. Bolt H. (2000). Carcinogenicity and genotoxicity of ethylene oxide: new aspects and recent advances. Crit. Rev. Toxicol. 30, 595–608. doi: 10.1080/10408440008951121, PMID: 11055837

[ref41] Tian F. Cai M. Li H. Qian Z. Chen L. Zou H.. (2022). Air pollution associated with incident stroke, Poststroke cardiovascular events, and death: a trajectory analysis of a prospective cohort. Neurology 99, e2474–e2484. doi: 10.1212/wnl.0000000000201316, PMID: 36171142

[ref42] Verhoeven J. Allach Y. Vaartjes I. Klijn C. de Leeuw F. (2021). Ambient air pollution and the risk of ischaemic and haemorrhagic stroke. Lancet Planet. Health 5, e542–e552. doi: 10.1016/s2542-5196(21)00145-5, PMID: 34390672

[ref43] Vincent M. Kozal J. Thompson W. Maier A. Dotson G. Best E.. (2019). Ethylene oxide: Cancer evidence integration and dose-response implications. Dose Response 17:155932581988831. doi: 10.1177/1559325819888317 PMC6906442 31853235

[ref9003] Viviane Z. R. Peter L. (2021). Obesity, inflammation, and atherosclerosis. Nat Rev Cardiol, 6, 399–409., PMID: 19399028 10.1038/nrcardio.2009.55

[ref44] Wu S. Yang Y. Zhu J. Wang L. Xu W. Lyu S.. (2024). Impact of hemoglobin adducts of ethylene oxide on the prevalence and prognosis of chronic kidney disease in US adults: an analysis from NHANES 2013-2016. Environ. Sci. Pollut. Res. Int. 31, 2802–2812. doi: 10.1007/s11356-023-30712-4, PMID: 38066258

[ref45] Xu Z. Xiangying K. Mengli C. Shi S. Iokfai C. Qingqing Z.. (2022). Blood ethylene oxide, systemic inflammation, and serum lipid profiles: results from NHANES 2013-2016. Chemosphere 299:134336. doi: 10.1016/j.chemosphere.2022.134336, PMID: 35337822

[ref46] Yixuan L. Nuozhou L. Wei X. Ruiyu W. (2024). Association between blood ethylene oxide levels and periodontitis risk: a population-based study. Front. Public Health 12:1338319. doi: 10.3389/fpubh.2024.1338319, PMID: 38384884 PMC10879552

[ref47] Zhang Y. Wang X. Li X. Lv S. Wang H. Liu Y.. (2023). Sirtuin 2 deficiency aggravates ageing-induced vascular remodelling in humans and mice. Eur. Heart J. 44, 2746–2759. doi: 10.1093/eurheartj/ehad381 37377116 10.1093/eurheartj/ehad381 PMC10393077

